# Annotations

**Published:** 1901-08-31

**Authors:** 


### ANNOTATIONS.

### Street Pavements.

August in London forciby reminds us of the great question of pavements which has puzzled mankind since the earliest periods. In small towns where the traffic is intermittent, the two factors which chiefly influence the authorities are cost and durability. In large cities many other considerations occur, of which noise, absorbent qualities, and the foothold of horses are all important. New pavements are invented every day, but it cannot be said that any one fulfils the ideal condition of a good foothold, a non-absorbent surface and little noise. Wood possesses the merit of deadening the sound of the traffic better than any other, and being usually covered with tar, is to a certain extent non-absorbent, but it has the great disadvantage that it easily cuts up and raises clouds of dust consisting of desiccating horse dung, tar, and particles of wood which is neither pleasant to inhale nor to catch in the corner of the eye. The varieties of asphalt do not cut up but are terribly slippery in certain weathers, and act as a veritable sounding shield for the noise of the traffic. Macadam is obviously unsuitable for any roads except those comparatively little used in the parks and the residential streets of the West End, for it cuts up very rapidly and raises clouds of metallic dust unless repeatedly watered. The latest form of pavement is the granolithic, which consists of blocks of an artificial composition of slag about the size of the ordinary wood blocks. These are laid on the same concrete base as the wood, but are separated from it by a layer of sand about one inch in thickness. The surface is non-absorbent, and is not so slippery as asphalt, but the noise is as great. The durability is supposed to be greater than any other form of pavement, but time only will prove this. The question of pavements will be immensely affected by the advent of the motor car, and it is just possible that in a few years we may witness the ideal condition of hard smooth non-absorbent pavements over which run rapid, noiseless and odourless machines. If this ideal condition is ever attained London and the larger towns will be the healthiest, the most pleasant, and the most convenient of all places in which to reside.

### Prisons, Workhouses, Convents, and Isolation Hospitals.

That bugs and fleas and other things unmentionable should hold high revel in one of the best known and most important of English jails is at least disgraceful, and yet from what has of late transpired such would seem to be the case at Holloway Prison. That there are difficulties in the way of securing cleanliness in a prison whose occupants are largely recruited from the scum of the population, is true, but that a great prison should have fallen into a verminous condition, and that the only excuse offered is that it "can't be helped," shows a disgraceful condition of affairs which must be rectified without delay. The matter, however, hardly ends there. These little creatures teach a lesson that must not be lost sight of—namely, to what an extent abuses may go and through what long periods they may continue in public institutions which are under lock and key, into which the light of publicity does not shine, and the "inspection" of which is of a purely official character. Prisons are not the only places which are under lock and key, nor the only places in which abuses may flourish merely because there is no one outside the official circle to insist on their iniquity. In workhouses the rule of the master is absolute and it is often the most difficult thing in the world to find out what is really going on within their walls. Who, again, is to ascertain with anything like exactness what is the inner life of those religious institutions of conventual type whose champions have lately succeeded in saving them from inspection even when their inmates are engaged in more or less dangerous trades. Then what about the little fever hospitals which are growing up all over the country? They are essentially "isolation" hospitals; children isolated from their friends are their chief inhabitants, and these children are all suffering from fevers in which delirium occurs, delirium which may stand as an explanation if strange tales are told. What places on this earth more demand careful and independent inspection than our smaller isolation hospitals, which have no resident medical staff, and whose patients are during the dark watches under the care of people who are in many cases by no means the élite of their profession? To be a member of the hospital sub-committee of some provincial sanitary authority may seem a small thing, an honour hardly to be sought for. But it is a post in which a man of energy and one who is not too ready to accept as gospel all that he is told may do his town the best of services. The price of liberty, we are told, is eternal vigilance, and if in an advanced civilisation we require institutions in which our fellows must be placed under the absolute control of officials, we must make provision for their proper inspection by non-official people. *Quis custodiet ipsos custodes?* In every little community there are a few inquisitive individuals who are voted by general acclaim to be most "objectionable people." They receive no thanks, yet they may be, and they often are, doing good service to their fellows by spying out the dark places of the land.

### Cheap Rides in Hot Weather.

We are told by the motorist that on those hot days which prevailed for so long and then so suddenly departed the only comfortable place was on a motor. Flying quickly through the air, with no sweltering horse in front to excite one's pity, there is the keen enjoyment of rapid movement, together with the stimulation of a pleasant breeze even on the stillest and most stagnant of autumn afternoons. So far, however, the motor is for the rich. Still for those of moderate purses there is the electric tram, and it is no bad substitute. Already in some of the outskirts of London people are finding out what has long been familiar to the inhabitants of New York and other American cities, namely, that a ride on a trolley-car in stifling August greatly conduces to good health. We are told that in New York on hot evenings the cars are loaded up with whole families taking their run into the country, and thus obtaining a blow of fresh air. And a writer recently describing the beauties of Jamaica aptly said that as in London the electric railway is called the "Twopenny Tube," so the Kingston electric cars might well be spoken of as the "Twopenny Fan," for they are open to the air on all sides and their speed is such that they afford a cool ride at all times, even in the middle of the day. And so with us. One has but to look at their passengers on the electric trams to see that the crowds who travel by them are not on business bent. Fresh air is their object, and no town wants this more than London.

